# Rapid diagnosis of *Mycoplasma pneumoniae* in children with pneumonia by an immuno-chromatographic antigen assay

**DOI:** 10.1038/srep15539

**Published:** 2015-10-21

**Authors:** Wei Li, Yujie Liu, Yun Zhao, Ran Tao, Yonggang Li, Shiqiang Shang

**Affiliations:** 1 Department of Laboratory, Children’s Hospital of Zhejiang University School of Medicine, Hangzhou 310003, P.R. China; 2 Hangzhou Genesis Biodetection & Biocontrol Ltd, Hangzhou 310018, P.R. China

## Abstract

*Mycoplasma pneumoniae* is a particularly important pathogen that causes community acquired pneumonia in children. In this study, a rapid test was developed to diagnose *M. pneumoniae* by using a colloidal gold-based immuno-chromatographic assay which targets a region of the P1 gene. 302 specimens were analyzed by the colloidal gold assay in parallel with real-time PCR. Interestingly, the colloidal gold assay allowed *M. pneumoniae* identification, with a detection limit of 1 × 10^3^ copies/ml. 76 samples were found to be positive in both real-time PCR and the colloidal gold assay; two specimens positive in real-time PCR were negative in the rapid colloidal gold assay. The specificity and sensitivity of the colloidal gold assay were 100% and 97.4%, respectively. These findings indicate that the newly developed immuno-chromatographic antigen assay is a rapid, sensitive and specific method for identifying *M. pneumoniae*, with potential clinical application in the early diagnosis of *Mycoplasma pneumoniae* infection.

*Mycoplasma pneumoniae* (*M. pneumoniae*) is one of the main pathogens that cause community-acquired pneumonia (CAP) in children^1^,^2^,^3^. It can trigger a series of pathophysiologic responses, which may cause respiratory tract symptoms (paroxysmal irritating cough, sore throat, sticky sputum, and/or purulent sputum) and various complications such as bronchial asthma, acute respiratory distress syndrome, Guillain-Barre syndrome, polyarthritis, and coronary artery diseases^4^,^5^,^6^. At present, several methods are available for the diagnosis of *M. pneumoniae* infection, including culture, serological tests, and real time PCR techniques^7^,^8^,^9^. However, *M. pneumoniae* culture and serological tests are insensitive, time-consuming and cross-reactive; therefore, they are not appropriate for rapid and accurate detection of *M. pneumoniae* infection in clinical practice^9^. Recently, real-time PCR has been reported by many authors as a rapid, sensitive, and specific method, but it still requires at least 2–4 hours for DNA extraction and amplification^10^,^11^,^12^,^13^. In addition, real-time PCR may not discriminate live *M. pneumoniae* from dead ones^9^. In this study, we developed a rapid test kit based on immuno-chromatography by using a pair of monoclonal antibodies which target the conserved region of the P1 surface protein of *M. pneumoniae*. Then, a large sample size study was conducted to assess the clinical diagnostic value of the newly developed system, in comparison with that of a commercial real-time PCR assay.

## Results

### *M. pneumoniae* detection in the colloidal gold assay

**Figure 1:**
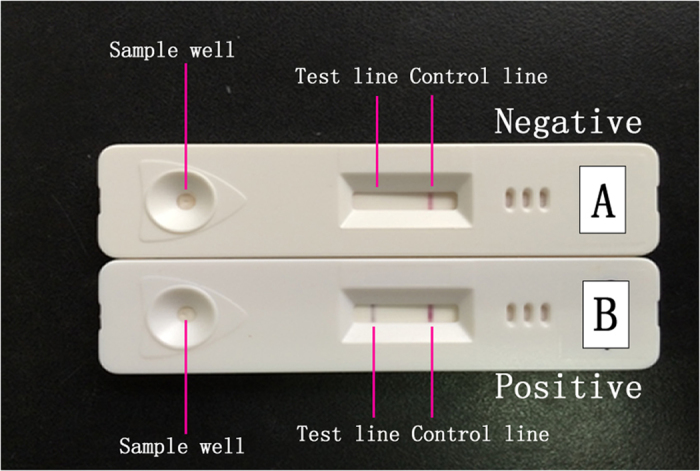
Colloidal gold plate setup (**A**) is representative of a negative sample, and (**B**) a positive sample).

**Figure 2:**
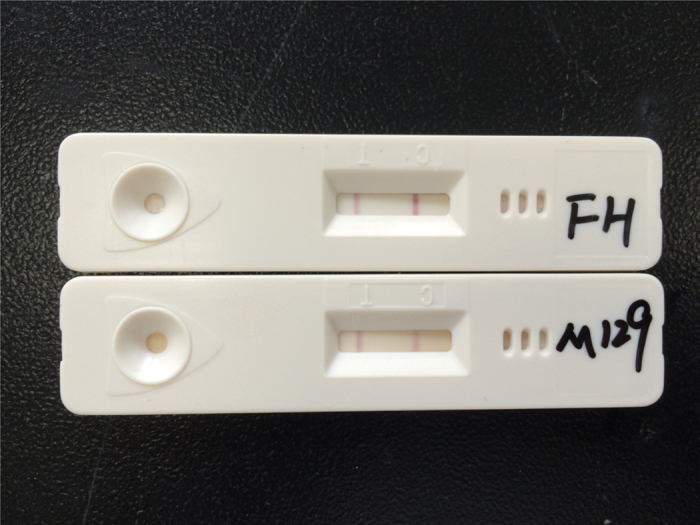
FH and M129 test results in the colloidal gold assay.

As shown in Fig. 1, *M. pneumoniae* presence in a sample resulted in both the test and control lines being positive. A sample without *M. pneumoniae* displayed only a positive control line. To confirm the detection capacity of the colloidal gold assay, P1 genes of standard types I and II *M. pneumoniae* strains were tested. The results showed that both FH (type II *M. pneumoniae)* and M129 (type I *M. pneumoniae)* strain were positive in the colloidal gold assay (Fig. 2).

### Sensitivity and specificity of the colloidal gold assay for *M. pneumoniae*

To evaluate the specificity of the colloidal gold assay, 10^5^ copies/ml of *M. pneumoniae, Staphylococcus aureus, Escherichia coli, Streptococcus pneumonia, Klebsiella pneumoniae, Legionella pneumophila, Haemophilus influenzae, Pseudomonas aeruginosa, anaerobic bacteria, Chlamydia trachomatis, Chlamydia pneumoniae*, adenovirus, respiratory syncytial virus, parainfluenza virus, influenza virus, human cytomegalovirus, human metapneumovirus and enteroviruses were assessed in 3 independent experiments. Our results showed that *M. pneumoniae* was identified correctly by the colloidal gold assay with no cross-reactions found between *M. pneumoniae* and other pathogens (Data not shown).

**Figure 3:**
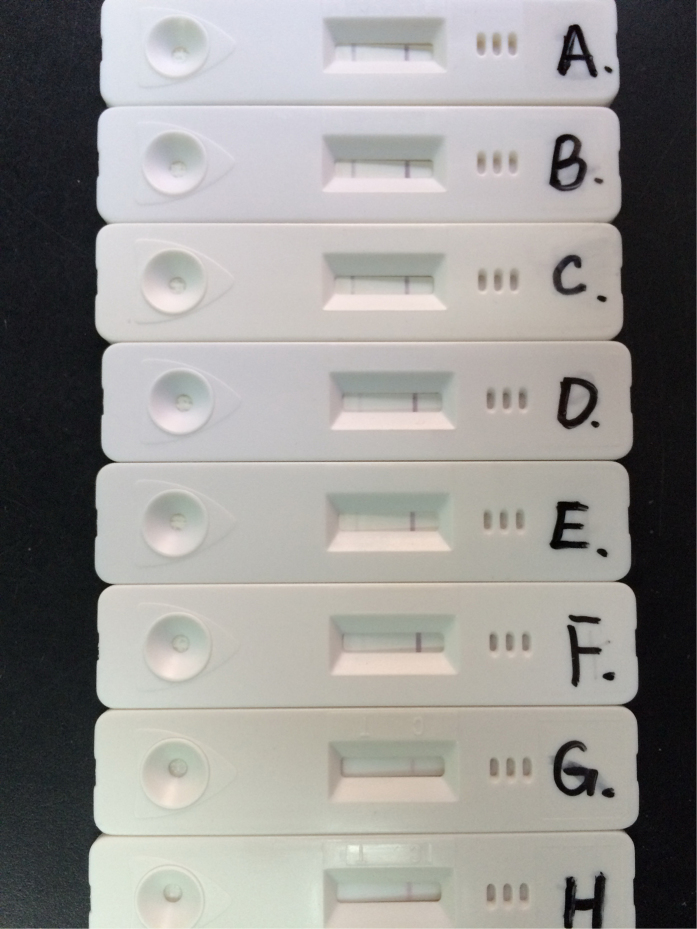
Sensitivity of the colloidal gold assay. (**A**) 10^7^ copies/ml; (**B**) 10^6^ copies/ml; (**C**) 10^5^ copies/ml; (**D**) 10^4^ copies/ml; (E) 10^3^ copies/ml; (**F**) 10^2^ copies/ml; (**G**) 10^1^ copies/ml; (**H**) Negative control.

To assess the sensitivity of the colloidal gold assay, standard *M. pneumoniae* was quantified by real-time PCR and submitted to serial 10-fold dilutions to obtain 10^1^ to 10^7^ copies/ml. Different concentrations of standard *M. pneumoniae* were tested by the colloidal gold assay. As shown in Fig. 3, *M. pneumoniae* with concentrations of 10^3^–10^7^copies/ml were positive in the colloidal gold assay (3 independent experiments). *M. pneumoniae* was not detected at 10^1^–10^2^ copies/ml.

### Clinical specimen data

A total of 78 throat swabs and 224 sputum specimens were collected from children with suspected *M. pneumoniae* infection. As shown in OTable 1, 78 (78/302, 25.8%) specimens were positive in real-time PCR. *M. pneumoniae* amounts ranged from 1 × 10^3^ to 8.65 × 10^7^ copies/ml. In the colloidal gold assay, 76 (76/302, 25.2%) samples were positive. The 76 specimens positive in the colloidal gold assay were also in real-time PCR; the corresponding patients were admitted to the hospital with a disease course of 5–10 days. There was a high statistical consistency (kappa value = 0.98, *p* = 0.000) between the colloidal gold assay and real-time PCR, indicating a high specificity for the newly developed method. Compared with real-time PCR, the specificity and sensitivity of the colloid gold assay were 100% and 97.4%, respectively. Only two samples were negative in the colloidal gold assay and positive in real-time PCR. Finally, *M. pneumoniae* DNA amounts in two samples were confirmed, with 1.3 × 10^3^ and 2.2 × 10^3^ copies/ml, respectively.

## Discussion

*M. pneumoniae* is a common pathogen of primary atypical pneumonia and other respiratory infectious diseases^14^,^15^. In addition, it is one of the most important agents of acute respiratory infections in children between the ages of 5 and 15 years^16^. Our previous study revealed a *M. pneumoniae* pneumonia infection rate of 18.5% in children of Hangzhou (China)^3^. A rapid and accurate method for diagnosing *M. pneumoniae* pneumonia is needed. Real-time PCR and serological tests are currently used to diagnose *M. pneumoniae* clinically in China^8^,^9^. But time-consuming and complicated protocols restrict their clinical application. Recently, Miyashita *et al.* reported a diagnostic sensitivity of only 60% that of real-time PCR for a commercial rapid antigen kit (Asahi Kasei Pharma Co., Tokyo, Japan)^20^. In this study, we applied colloidal gold assay to detect *M. pneumoniae* by targeting the specific P1 antigen. P1 is one of the major surface proteins of M*. pneumoniae* and its gene is an attractive target in the clinical detection of M*. pneumoniae* by real-time PCR^10^,^17^. This is the first study detecting P1 antigen to confirm *M. pneumoniae* infection in children with pneumonia. Clinical *M. pneumoniae* strains can be divided into two types (I and II) according to their P1 gene variants^18^. Colloidal gold assay has high capacity to detect both types of *M. pneumoniae. M. pneumoniae* detection could be completed in 15 minutes by using our method, while real-time PCR requires 2 to 4 hours, and culture usually takes 21 days^19^. Moreover, this method presents high sensitivity and specificity in detecting *M. pneumoniae* (no cross-reactions with other clinical pathogens), while serological tests often show lower specificity^9^. Of note, we found 10^3^ copies/ml was the detection limit for this new method, while this value is 8.3 × 10^4^ copies/ml for the Asahi company rapid antigen kit^20^.

In clinical practice, we used commercial real-time PCR assay as a control method, targeting the P1 gene of *M. pneumoniae*. We applied the newly developed colloidal gold assay to the 302 specimens from children with pneumonia. 76 (25.8%) specimens were positive for *M. pneumoniae*. When compared with real-time PCR, the specificity and sensitivity of the colloidal gold assay were 100% and 97.4%, respectively. The symptoms in all *M*. pneumoniae positive children were improved after treatment with Azithromycin. Two samples were positive in real time PCR but negative in the colloidal gold assay. Real-time PCR and clinical data were obtained from the two specimens, which both had 10^3^ copies/ml. It should be noted that these 2 patients were treated with antibiotics for more than a week before visiting our hospital. We hypothesize that the two samples may have only contained low amounts of live *M. pneumoniae*, below the detection limit of the colloidal gold assay.

In conclusion, the colloidal gold assay is a rapid, sensitive, and specific method for the identification of *M. pneumoniae*. Most importantly, this method is easy to operate without any special instrument and may be suitable for bedside detection. These findings indicate that the newly developed colloidal gold assay would be an effective method for detecting *M. pneumoniae* in clinical practice.

## Methods

### *M. pneumoniae* strain and control strains

Standard *M. pneumoniae* FH (ATCC 15531) and M129 (ATCC 29342) strains were purchased from ATCC. Negative controls used in this study included *Staphylococcus aureus, Escherichia coli, Streptococcus pneumonia, Klebsiella pneumoniae, Legionella pneumophila, Haemophilus influenzae, Pseudomonas aeruginosa, anaerobic bacteria, Chlamydia trachomatis, Chlamydia pneumoniae*, adenovirus, respiratory syncytial virus, parainfluenza virus, influenza virus, human cytomegalovirus, human metapneumovirus and enteroviruses. All negative controls were isolated from patients and conserved at the Department of clinical laboratory, Children’s Hospital of Zhejiang University School of Medicine.

### Clinical specimens from children with pneumonia

From February to August 2014, 302 children were enrolled in this study. The inclusion criteria were: (1) age < 14 years; (2) patient visiting the Children’s Hospital of Zhejiang, University School of Medicine; (3) primary diagnosis as pneumonia according to known guidelines^21^. During six months, 302 specimens were collected, including 78 throat swabs and 224 sputum samples in our hospital. Each specimen was mixed with 1.5 ml saline and stored at −70 °C; 1 ml of the mixture was used for real-time PCR and 0.5 ml in the colloidal gold assay. The 302 patients (125 females and 177 males) were 3 months to 10 years old.

The study was performed in accordance with the Declaration of Helsinki and approved by the Medical Ethics Committee of Zhejiang University School of Medicine. All patients provided informed consent.

### Preparation of the colloidal gold plate

As shown in Fig. 1, the colloidal gold plate contained three parts: 1) sample well; 2) reagent region; 3) chromatography region. The reagent region contained monoclonal antibody A (mouse) labeled with colloidal gold which targets the *M. pneumoniae* P1 antigen. The chromatography region included *M. pneumoniae* monoclonal antibody B (mouse) which is bound to the Test line position and mouse IgG polyclonal antibody bound to the Control line position in the chromatography. A sample with *M. pneumoniae* would result in monoclonal antibody A binding with *M. pneumoniae* and detected by monoclonal antibody B as well as mouse IgG polyclonal antibody (both of Test and Control lines positive). In a sample without *M. pneumoniae*, only monoclonal antibody A can be detected by IgG polyclonal antibody (Test line negative and Control line positive). The plate was manufactured by Genesis Biodetection & Biocontrol Ltd, Hangzhou, China.

### Real-time PCR for *M. pneumoniae* detection

For real-time PCR, 1 ml of the mixture was transferred into a 1.5-ml microcentrifuge tube aseptically and centrifuged for 5 min at 12000 rpm/min. The cell pellets were resuspended in 50 μl lysis buffer (Da An Gene Co., Ltd., China); 4 μl lysate served as template in real-time PCR amplification based on the TaqMan probe PCR kit (Da An Gene Co., Ltd., China) as reported previously^3^,^22^. For each assay, negative and positive quality controls, and four positive quantity plasmid controls (10^4^, 10^5^, 10^6^, and 10^7^ copies/ml) were assessed. Ct values of the four quantity controls were then subjected to log-linear analysis to generate a standard curve used to determine the concentrations of clinical *M. pneumoniae* samples. Real-time PCR was carried out on an ABI 7500 instrument for 3 min at 95 °C, followed by 40 two-step cycles (15 s at 95 °C and 45 s at 55 °C).

### Colloidal gold assay for *M. pneumoniae* detection

To perform the immune-chromatographic assay, 100 μl (about 3 drops) of the mixture was added into a sample well for 10–15 minutes. Samples with positive control and test lines were determined as *M. pneumoniae* positive; no Control line on the plate indicated an invalid test, and a second test plate was used till the result was either positive or negative.

### Statistics

Statistical analysis was performed using the kappa test; statistical significance was calculated using the SPSS 17.0 software (SPSS Inc., Chicago, IL, USA).

## Additional Information

**How to cite this article**: Li, W. *et al.* Rapid diagnosis of *Mycoplasma pneumoniae* in children with pneumonia by an immuno-chromatographic antigen assay. *Sci. Rep.* **5**, 15539; doi: 10.1038/srep15539 (2015).

## Figures and Tables

**Table 1:** Comparison of detection results between the colloidal gold assay and real-time PCR.

**Colloidal gold assay**	**Real-time PCR**		**Total**
	**Positive**	**Negative**	
Positive	76	0	76
Negative	2	224	226
Total	78	224	302
